# The Molecular Basis of Ligand Interaction at Free Fatty Acid Receptor 4 (FFA4/GPR120)*

**DOI:** 10.1074/jbc.M114.561449

**Published:** 2014-05-24

**Authors:** Brian D. Hudson, Bharat Shimpukade, Graeme Milligan, Trond Ulven

**Affiliations:** From the ‡ Molecular Pharmacology Group, Institute of Molecular, Cell and Systems Biology, College of Medical, Veterinary and Life Sciences, University of Glasgow, Glasgow G12 8QQ, Scotland, United Kingdom and; the § Department of Physics, Chemistry and Pharmacy, University of Southern Denmark, Campusvej 55, DK-5230 Odense M, Denmark

**Keywords:** 7-Helix Receptor, Bioluminescence Resonance Energy Transfer (BRET), Fatty Acid, G Protein-coupled Receptor (GPCR), Homology Modeling, Diabetes

## Abstract

The long-chain fatty acid receptor FFA4 (previously GPR120) is receiving substantial interest as a novel target for the treatment of metabolic and inflammatory disease. This study examines for the first time the detailed mode of binding of both long-chain fatty acid and synthetic agonist ligands at FFA4 by integrating molecular modeling, receptor mutagenesis, and ligand structure-activity relationship approaches in an iterative format. In doing so, residues required for binding of fatty acid and synthetic agonists to FFA4 have been identified. This has allowed for the refinement of a well validated model of the mode of ligand-FFA4 interaction that will be invaluable in the identification of novel ligands and the future development of this receptor as a therapeutic target. The model reliably predicted the effects of substituent variations on agonist potency, and it was also able to predict the qualitative effect of binding site mutations in the majority of cases.

## Teaser

**Background:** FFA4 is a receptor for long-chain fatty acids and is considered a novel target for metabolic diseases.

**Results:** Combinations of molecular modeling, receptor mutagenesis, and ligand structure-activity relationships defined the binding pocket.

**Conclusion:** Fatty acid and synthetic agonists share an overlapping binding site.

**Significance:** The validated homology model will assist the search for novel ligands.

## Introduction

FFA4 (previously designated GPR120) is a 7-transmembrane G protein-coupled receptor (GPCR)^4^ activated by long-chain fatty acids (LCFAs) that has been receiving increasing interest in recent years as a novel therapeutic target for the treatment of metabolic conditions, including diabetes (1, 2). This is in large part based on studies demonstrating that activation of FFA4 by LCFA ligands results in a number of potentially beneficial biological effects, including stimulation of secretion of glucagon-like peptide-1 from enteroendocrine cells (3), inhibition of ghrelin secretion (4), stimulation of glucose uptake by adipocytes (5), promotion of pancreatic β-cell survival (6), and inhibition of the release of pro-inflammatory cytokines from macrophages that, in turn, improve systemic insulin sensitivity (5). In addition, genetic studies have shown that disruption of FFA4 function in both mice and humans is linked to obesity (7). Taken together, these observations suggest that FFA4 agonists represent an exciting new possibility for the treatment of metabolic disease.

Despite the recent interest in FFA4, its development as a therapeutic target has been hindered by a lack of available ligands and, in particular, a lack of ligands with suitable selectivity for FFA4 over FFA1, the other GPCR activated by LCFAs. Indeed, beyond the use of LCFAs that activate FFA1 and FFA4 with very similar potency, much of the early work on FFA4 utilized the compound GW9508, despite the fact that this ligand has only modest potency at FFA4 (1–5 μm) and is ∼100-fold more potent at FFA1 (8). Early attempts to identify novel FFA4-selective agonists yielded only modest success, with all compounds described displaying low potency and/or very limited selectivity for FFA4 over FFA1 (9, 10). However, the recent description of *ortho*-biphenylmethoxyphenylpropionic acid derivatives as selective FFA4 agonists, as exemplified by the most potent and selective compound TUG-891, has provided a means to selectively probe the function of FFA4 (11). Indeed, we have recently used TUG-891 to explore in detail the *in vitro* pharmacology of FFA4 to begin to define the therapeutic potential of this receptor (12).

Although TUG-891 currently represents the best available FFA4-selective ligand, this compound still possesses relatively modest potency and high lipophilicity (*C*log*P* = 5.8), and it has been shown to have limited selectivity for the mouse ortholog of FFA4 over mouse FFA1 (12). Considering these factors, there is a clear need to identify novel FFA4 agonists. Structure-based ligand design represents one approach that might be used to identify ligands for this receptor. However, although this approach has been successfully used for other GPCRs (13, 14), the lack of atomic level structural information on FFA4 is currently a restriction. Although there have been great advances in recent years within the field of GPCR structural biology (15), it still remains impractical to obtain high resolution structures for receptors on demand. Instead, structure-based drug design must utilize homology modeling of the receptor and its binding site linked to validation by detailed receptor mutagenesis and functional studies.

In this study, a combination of molecular modeling, mutagenesis, and ligand structure-activity relationship (SAR) studies have been employed, through an iterative approach, to define the orthosteric binding site of FFA4. Through this, we have identified key residues within FFA4 involved in binding of the LCFA, α-linolenic acid (aLA), the previously studied FFA1/FFA4 agonist GW9508, as well as TUG-891. We demonstrate key differences in the interaction of each ligand with FFA4 and provide rationale for the high potency of the structural scaffold of TUG-891. Furthermore, examining SAR variations of this *ortho*-biphenyl scaffold has identified key residues in FFA4 responsible for the preference for the terminal methyl substituent present in TUG-891. Taken together, combining these approaches has allowed us to develop a well validated model of ligand interaction with FFA4 that will be useful in the future identification of novel ligands for this receptor.

## EXPERIMENTAL PROCEDURES

### 

#### 

##### Materials

Compounds TUG-670, TUG-804, TUG-826, TUG-827, TUG-839, TUG-853, TUG-854, TUG-856, and TUG-891 were synthesized as described previously (11). aLA and GW9508 (4-[[(3-phenoxyphenyl)methyl]amino]benzenepropanoic acid) were purchased from Sigma. Tissue culture reagents were from Invitrogen. Molecular biology enzymes and reagents were from Promega. All other experimental reagents were from Sigma.

TUG-909 (3-(4-((4-fluoro-3′-methyl-[1,1′-biphenyl]-2-yl)methoxy)phenyl)propanoic acid) was synthesized as follows. A flask containing *m*-tolylboronic acid (35 mg, 0.25 mmol), methyl 3-(4-((2-bromo-5-fluorobenzyl)-oxy)-phenyl)propanoate (10) (85 mg, 0.23 mmol), potassium carbonate (38 mg, 0.28 mmol), acetonitrile (1.75 ml), water (0.18 ml), and Pd(PPh_3_)_4_ (14 mg, 0.012 mmol) under argon was heated at 80 °C for 20 h. The reaction was cooled to room temperature, concentrated, diluted with ethyl acetate, and filtered. The filtrate was washed with water and brine, dried (Na_2_SO_4_), and concentrated. The residue was purified by flash chromatography (SiO_2_, EtOAc/petroleum ether, 1:9) to give 64 mg (73%) of the methyl ester of TUG-909 as a colorless oil: *R_f_* = 0.50 (EtOAc/petroleum ether, 1:2); ^1^H NMR (400 MHz, CDCl_3_) δ 7.34 (dd, *J* = 9.7, 2.7 Hz, 1H), 7.31–7.24 (m, 2H), 7.19–7.02 (m, 6H), 6.80–6.74 (m, 2H), 4.89 (s, 2H), 3.65 (s, 3H), 2.87 (t, *J* = 7.8 Hz, 2H), 2.58 (t, *J* = 7.8 Hz, 2H), 2.35 (s, 3H); ^13^C NMR (100 MHz, CDCl_3_) δ 173.5, 162.4 (d, *J_CF_* = 246.1 Hz), 157.0, 139.6, 138.2, 136.8 (d, *J_CF_* = 7.5 Hz), 133.2, 131.6 (d, *J_CF_* = 8.0 Hz), 130.1, 129.4, 128.4 (d, *J_CF_* = 4.3 Hz), 126.3, 115.4 (d, *J_CF_* = 22.4 Hz), 115.1, 114.8 (d, *J_CF_* = 21.1 Hz), 67.8, 51.7, 36.1, 31.1, 30.2, 21.6.

The methyl ester of TUG-909 (20 mg, 0.053 mmol) in THF (0.27 ml) was added to LiOH·H_2_O (7.0 mg, 0.16 mmol) in water (0.32 ml). The reaction mixture was stirred at room temperature for 4 h, then acidified with 1 m HCl solution until pH < 1, and extracted with ethyl acetate. The combined organic phase was washed with brine, dried (Na_2_SO_4_), and concentrated to give 19 mg (98%) of TUG-909 as a white solid: mp 94–95 °C; *R_f_* = 0.10 (EtOAc/petroleum ether, 1:2); ^1^H NMR (400 MHz, DMSO-*d*_6_) δ 12.17 (br s, 1H), 7.42–7.15 (m, 7H), 7.10 (d, *J* = 8.1 Hz, 2H), 6.78 (d, *J* = 8.3 Hz, 2H), 4.89 (s, 2H), 2.78–2.71 (m, 2H), 2.58 to 2.41 (m, 2H), 2.28 (s, 3H); ^13^C NMR (100 MHz, DMSO-*d*_6_) δ 172.2, 161.4 (d, *J_CF_* = 244.0 Hz), 156.2, 138.8, 137.8 (d, *J_CF_* = 3.0 Hz), 136.6 (d, *J_CF_* = 7.6 Hz), 133.4, 132.9, 131.8 (d, *J_CF_* = 8.1 Hz), 129.7, 129.2, 128.2, 128.1 126.0, 115.6 (d, *J_CF_* = 21.9 Hz), 114.9 (d, *J_CF_* = 20.9 Hz), 114.53, 67.1, 35.3, 29.4, 20.9; HRMS calculated for C_23_H_21_FO_3_Na (M + Na^+^) was 387.1367 and found was 387.1378.

##### Homology Modeling

The sequences of the short isoform of human FFA4 and the nanobody-stabilized β_2_-adrenoreceptor (Protein Data Bank 3P0G) (16) were aligned manually. Homology models of FFA4 were constructed using the Prime module implemented in the Schrödinger suite 2012 (17). The FFA4 model was built using default settings, preprocessed using the OPLS-2005 force field, added hydrogen atoms, and assigned partial charges. Hydrogen bond assignment was done at pH 7.4 using PROPKA (18). Restrained minimization was performed until heavy atoms converged to root mean square deviation = 0.3 Å using the OPLS-2005 force field. Loop refinement was performed using Prime's loop refinement module (17). For short loops, the default sampling rate was chosen, whereas the highest sampling rate was used for loops with more than eight amino acid residues. Variable dielectric surface-generalized Born model was selected as the solvent model. Side chains within 7 Å of the loop were energy-minimized with the energy cutoff at 10 kcal/mol. Mutations were introduced in the hFFA4 model in Maestro, and rotamers were adjusted manually to minimize side-chain clashes. The mutant models were preprocessed and restrained minimized using the OPLS-2005 force field in the Protein Preparation Wizard.

##### Ligand Preparation and Docking

All ligands were converted to three-dimensional structures in Maestro. Macromodel was used for energy minimization of ligands using the OPLS-2005 force field (19). Ligands were prepared using LigPrep (39). Ionization states were generated using Epik at pH 7.0 ± 1.0, and low energy ring conformations were restricted to one per ligand (20). Induced-fit docking studies were performed using the IFD 2006 protocol as implemented in Schrödinger suite 2013-1 (21). The docking box was placed to include Arg-99^2.64^, Phe-115^3.29^, Phe-211^5.42^, Trp-277^6.48^, and Phe-304^7.36^. Hydrogen bond constraints were set between the carboxylate of the ligand and Arg-99^2.64^ and applied in both docking stages. Ligand conformational sampling was performed using default settings. Initial Glide docking was performed using standard settings, and the maximum number of poses per ligand was restricted to 15. Prime was used to refine residues within 5.0 Å of ligand poses. Re-docking was performed in Glide using the extra precision mode for the 10 highest ranking protein-ligand complex structures generated in the initial docking, which was within 30.0 kcal/mol of the lowest energy protein-ligand complex structure.

##### Calculation of Ligand Binding Energies

Molecular mechanics with generalized Born and surface area solvation (MM-GBSA) simulations were used to calculate ligand-binding energy (Δ*G*_bind_) (22). Calculations were performed for all the docking poses resulting from induced-fit docking. Complexes were taken from separated ligand and protein structures; the variable dielectric surface-generalized Born solvation method was used, and the protein flexibility region was restricted to 4.0 Å from ligand. In sampling, only side chains were minimized, and constraints were applied on flexible residues to restrict them from deviating too much from their initial position.

##### Plasmids and Mutagenesis

Plasmids encoding the short isoform of human FFA4 fused at its C terminus to enhanced yellow fluorescent protein (eYFP) and containing an N-terminal FLAG epitope tag and β-arrestin-2 fused to *Renilla* luciferase (Rluc) were as described previously (11, 12). Mutations were introduced into the FFA4 sequence using the QuikChange method (Stratagene), and in all cases the presence of the mutation was verified through sequencing.

##### Generation of Stable Inducible Cell Lines

Stable inducible 293 cell lines were generated for either wild type or mutant FFA4-eYFP constructs using the Flp-In^TM^ T-REx^TM^ system (Invitrogen). Briefly, Flp-In^TM^ T-REx^TM^ 293 cells were co-transfected with the desired FFA4-eYFP construct and the pOG44 plasmid encoding the Flp recombinase enzyme. This allows for polyclonal selection of cells using hygromycin B to generate cell lines with inducible expression of the desired receptor in response to the antibiotic doxycycline. Once generated, receptor expression and doxycycline sensitivity were confirmed by fluorescence microscopy to detect the C-terminal eYFP tag.

##### β-Arrestin-2 Interaction Assay

HEK293T cells were maintained in Dulbecco's modified Eagle's medium supplemented with 10% heat-inactivated FBS at 37 °C and 5% CO_2_. Transfections were carried out with polyethyleneimine, and experiments were conducted 48 h post-transfection according to a previously described protocol (12). Briefly, HEK293T cells were co-transfected with FLAG-FFA4-eYFP (or appropriate mutant) and β-arrestin-2-Rluc plasmids in a ratio of 4:1. 24 h post-transfection, cells were subcultured into poly-d-lysine-coated white 96-well tissue culture plates and maintained for a further 24 h prior to the experiment. To conduct the experiments, cells were first washed and then incubated at 37 °C for 30 min in Hanks' balanced salt solution. The Rluc substrate coelenterazine h was added to a final concentration of 2.5 μm before a further incubation for 10 min at 37 °C. Test compounds were then added at the specified concentration, and cells were incubated for a final 5-min period prior to measuring luminescent emission at 530 and 490 nm using a PheraStar FS plate reader (BMG Lab Tech) fitted with a BRET1 optic module. The 530/490 emission ratio was calculated and corrected for the ratio obtained in cells transfected with only the β-arrestin-2-Rluc plasmid to obtain the “net BRET” signal. To compare the magnitude of signal across FFA4 mutants, net BRET values were normalized against the signal obtained from wild type FFA4 treated with 100 μm aLA.

##### Total and Cell Surface Receptor Expression Measurements

HEK293T cells co-transfected with FLAG-FFA4-eYFP (or mutant) and β-arrestin-2-Rluc plasmids in a 4:1 ratio were subcultured 24 h after transfection into poly-d-lysine-coated black 96-well tissue culture plates with clear bottoms. After 24 h, cells were incubated with an anti-FLAG monoclonal primary antibody diluted in culture medium for 30 min at 37 °C. Cells were washed with phosphate-buffered saline (PBS) and then incubated for 30 min with a combination of Hoechst 33342 and a horseradish peroxidase-conjugated anti-mouse IgG secondary antibody. Cells were then washed three times with PBS before reading both eYFP (excitation 500 nm and emission 535 nm) and Hoechst (excitation 355 nm and emission 460 nm) on a PolarStar Omega plate reader (BMG LabTech). Cells were then washed a final time with PBS before incubation in the dark with the horseradish peroxidase SureBlue^TM^ TMB substrate (KPL, Inc.). Absorbance at 620 nm was then measured on a PolarStar Omega plate reader. To obtain total expression, the eYFP fluorescence was corrected for cell number based on the Hoechst fluorescence. Surface expression was reported as the 620 nm absorbance corrected for cell number based on Hoechst fluorescence. In both cases, all values were expressed as a percentage of the signal obtained from wild type FLAG-FFA4-eYFP-transfected cells.

##### Ca^2+^ Mobilization Assays

Flp-In^TM^ T-REx^TM^ 293 cells engineered to express the desired form of FFA4 were plated 50,000 cells/well in black 96-well plates with clear bottoms. Cells were then treated with 100 ng/ml doxycycline to induce receptor expression and maintained overnight at 37 °C and 5% CO_2_ prior to their use. For the assay, cells were first pre-labeled for 45 min with the calcium-sensitive dye Fura2-AM, then washed, and maintained in Hanks' balanced salt solution. Fura-2 fluorescent emission at 510 nm resulting from 340 or 380 nm of excitation was then monitored using a Flexstation II plate reader (Molecular Devices). Basal fluorescence was measured for 16 s; test compounds were then added, and fluorescence was measured for an additional 74 s. The background subtracted peak 340/380 ratio obtained following compound addition was then used to plot concentration-response data.

##### Data Analysis and Curve Fitting

All data presented represent the means ± S.E. of at least three independent experiments. Data analysis and curve fitting were carried out using the GraphPad Prism software package version 5.0b. Concentration-response data were plotted on a log axis, where the untreated vehicle control condition was plotted at 1 log unit lower than the lowest test concentration of ligand and fitted to a three-parameter sigmoidal concentration-response curve. Statistical analysis was carried out using one-way analysis of variance and Bonferroni post hoc test.

## RESULTS

### 

#### 

##### Generating and Testing an Initial FFA4 Receptor Model and Selecting Residues for Mutagenesis

To define the ligand binding pocket of human FFA4 and to identify specific residues important for ligand interaction, we employed combinations of molecular modeling, receptor mutagenesis, and ligand SAR studies. Previous work defining the mode of binding of orthosteric agonists to the free fatty acid receptors FFA1–FFA3 has demonstrated that a pair of Arg residues near the top of the transmembrane (TM) helical bundle, at positions 5.39 and 7.35 (Ballesteros and Weinstein GPCR numbering scheme (23)), form critical ionic interactions with the carboxylate of both endogenous fatty acids and various synthetic ligands (24, 25). However, FFA4 is only distantly related to FFA1–FFA3, and these two positively charged residues are not conserved. Instead, several studies have implicated a single Arg at position 2.64 (amino acid 99 in the primary sequence) as the critical residue involved in the interaction between FFA4 and the carboxylate of its ligands (12, 26, 27). Based on this key interaction, we developed models of the FFA4 ligand binding pocket, including one described previously (11), to predict additional residues that might be involved in binding to the receptor of either the endogenous agonist aLA or selected synthetic ligands. From this, 21 residues predicted to contribute to or to be in close proximity to the ligand binding pocket were identified and subjected to Ala replacement (except for R99Q^2.64^ and F303H^7.35^) mutagenesis (Table 1, top).

**TABLE 1:**
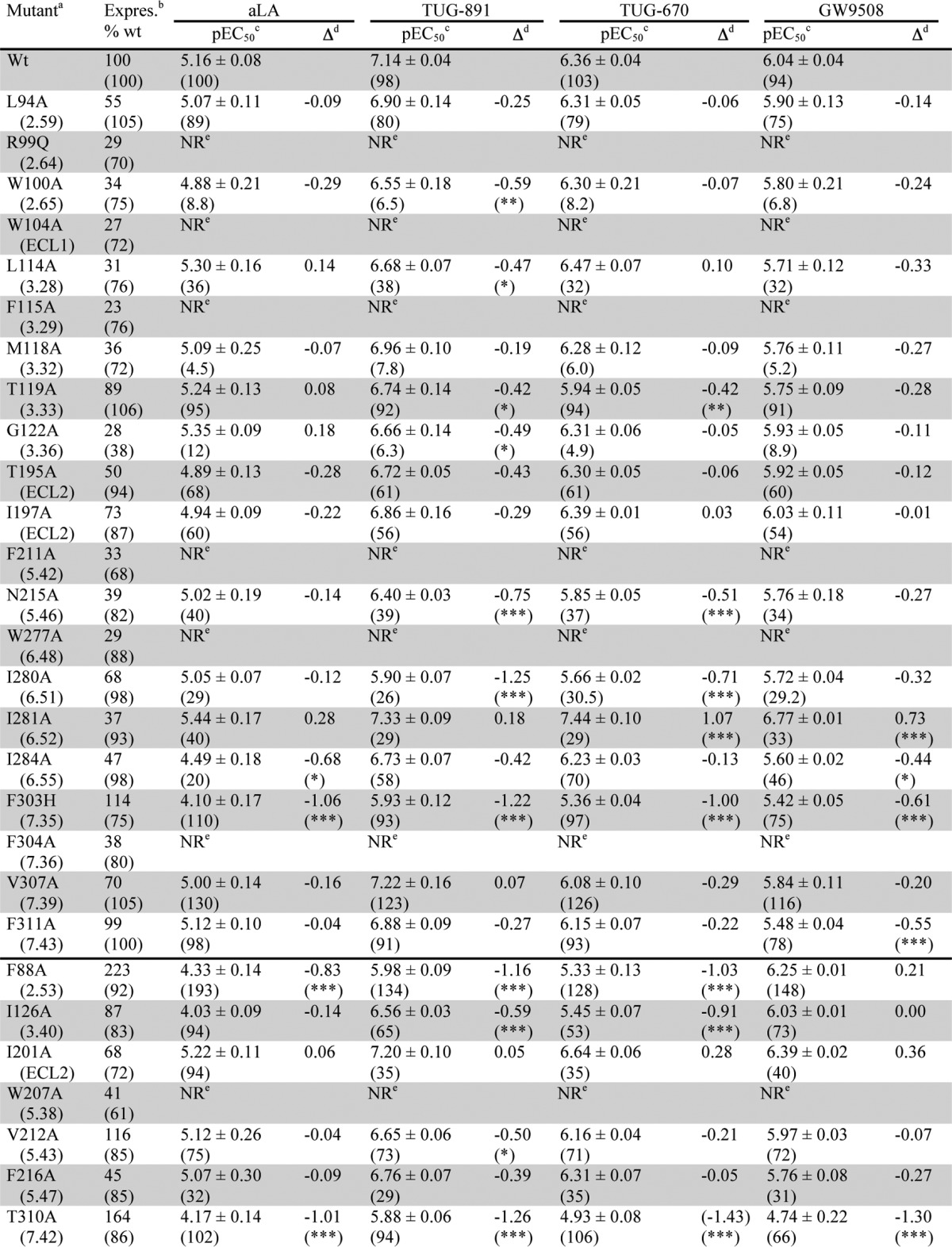
**Potency and efficacy in a β-arrestin-2 recruitment assay of aLA, TUG-891, TUG-670, and GW9508 on wild type FFA4 and mutations predicted to be in close proximity to the ligand binding pocket** NR means no response.

*^a^* Primary amino acid residue number with Ballesteros and Weinstein position in parentheses.

*^b^* Cell surface expression is shown as a percentage of wild type; total expression is in parentheses.

*^c^* pEC_50_ values with efficacy expressed as a percentage of the wild type aLA response in parentheses.

*^d^* (pEC_50_ mutant) − (pEC_50_ WT FFA4). Statistical significance is as follows: +, *p* < 0.05; ++, *p* < 0.01; +++, *p* < 0.001.

##### Assessment of the Initial Mutants

**FIGURE 1.**
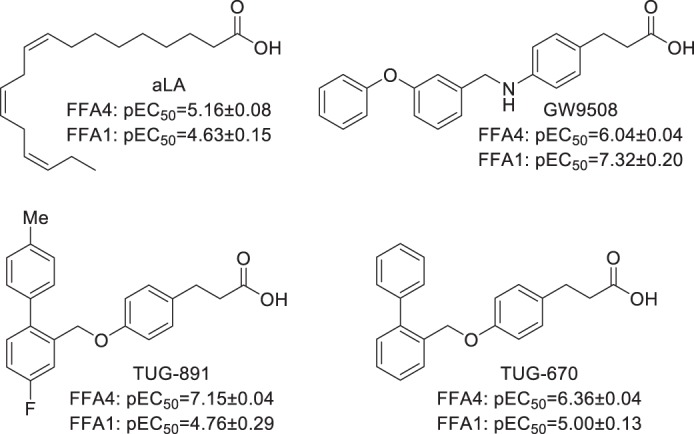
**Structures and potencies of ligands used in this study at human FFA4 and FFA1.** Potencies were determined using the β-arrestin-2 recruitment assay.

To assess the impact of the 21 initial mutations, aLA and three synthetic ligands were chosen for analysis (Fig. 1). These were the potent and selective FFA4 agonist TUG-891; the analog TUG-670, lacking the 4′-methyl and 4-fluoro substituents that enhance potency and selectivity at FFA4; and GW9508, an FFA1/FFA4 agonist that has been widely used in FFA4 studies, despite being markedly selective for FFA1 (5, 8, 12). Because direct ligand binding assays are not currently available for FFA4, a BRET-based FFA4-β-arrestin-2 interaction assay was used as the primary assay to assess the effect of FFA4 mutations on ligand-receptor interactions (Table 1). This assay was chosen because the ligand-stimulated GPCR-β-arrestin-2 interaction is predicted to occur in a 1:1:1 (ligand/GPCR/β-arrestin-2) molecular ratio, and therefore ligand potency in this assay is anticipated to provide a very good surrogate measure of ligand binding affinity (28). However, to ensure that the identified FFA4 binding pocket did not reflect a β-arrestin-2 biased receptor conformation, results with key mutants were also confirmed in Ca^2+^ mobilization assays (Table 2) that represent a G_q/11_-mediated FFA4 signaling end point. Comparing the effects of the four test ligands at wild type FFA4 in the β-arrestin-2 and Ca^2+^ assays (Tables 1 and 2) demonstrated the same rank order of potency in each assay such that TUG-891 > TUG-670 > GW9508 > aLA; however, it was apparent that in the Ca^2+^ assay system the measured potency values for the compounds were generally lower than observed in the β-arrestin-2 assay. Initial testing with the β-arrestin-2 assay indicated that all four ligands were inactive at an R99Q^2.64^ mutant of FFA4 (Table 1), suggesting that all appear likely to bind to the orthosteric site of the receptor.

**TABLE 2:**
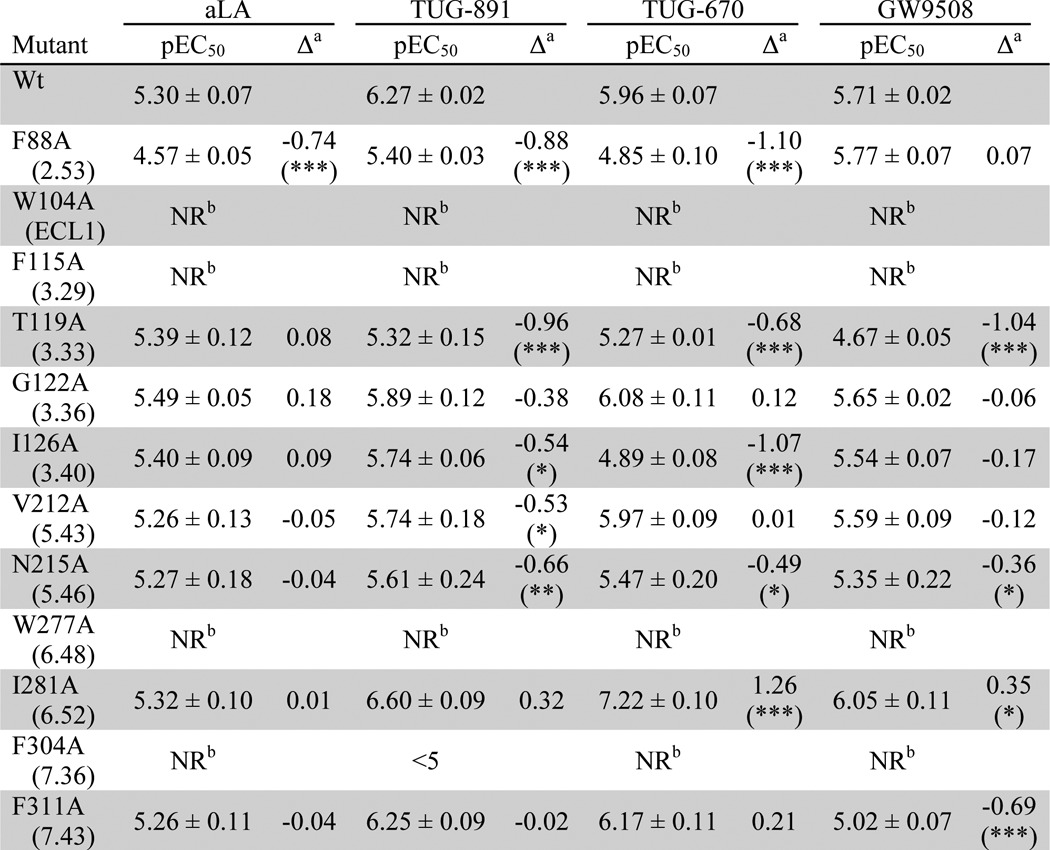
**Potency of aLA, TUG-891, TUG-670, and GW9508 on wild type FFA4 and selected mutants in a Ca^2+^ mobilization assay** NR means no response.

*^a^* (pEC_50_ mutant) − (pEC_50_ WT FFA4). Statistical significance is as follows: +, *p* < 0.05; ++, *p* < 0.01; +++, *p* < 0.001.

**FIGURE 2.**
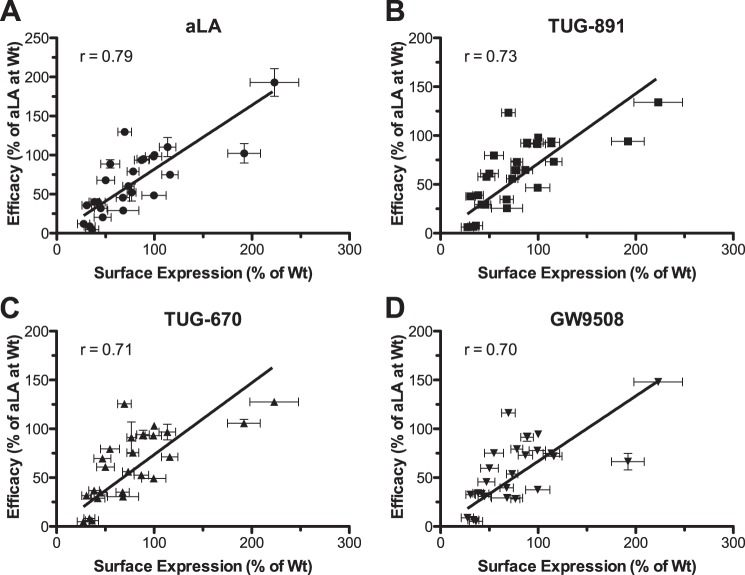
**Cell surface expression is correlated with β-arrestin-2 response efficacy for FFA4 mutants.** Correlation plots of receptor-β-arrestin-2 BRET response efficacy *versus* measured cell surface expression are shown for aLA (*A*), TUG-891 (*B*), TUG-670 (*C*), and GW9508 (*D*). Mutants that gave no ligand-mediated response were excluded from these analyses. Correlation coefficients are also shown.

Analysis of the other 20 initial mutations (Table 1, top) indicated that all were expressed when transfected with total expression levels ranging between 38 and 106% of wild type FFA4 levels. Each mutant was also successfully delivered to the surface of the cell; however, for most the cell surface expression was lower than the total expression, suggesting that these mutants did not fold and traffic to the cell surface as efficiently as the wild type receptor. Despite this, cell surface expression correlated significantly with β-arrestin-2 recruitment response efficacy (*p* < 0.001), yielding correlation coefficients of 0.79, 0.73, 0.71, and 0.70 for aLA, TUG-891, TUG-670, and GW9508, respectively (Fig. 2, *A–D*). Although only weaker correlations were observed (0.44, 0.58, 0.62, and 0.51, for aLA, TUG-891, TUG-670, and GW9508, respectively) between total receptor expression and response efficacy, this was anticipated as the β-arrestin-2 recruitment assay measures only activation of receptors expressed at the cell surface. The strong correlation observed between response efficacy and cell surface expression suggests that the majority of efficacy variations observed among the mutants result from expression differences and not because these mutations alter the ability of FFA4 to be activated. Importantly, although variations in ligand efficacy were associated with mutant expression levels, this should not be the case for ligand potency, due to the 1:1:1 ratio of ligand/receptor/β-arrestin-2. In accordance with this, the fact that increased β-arrestin-2 efficacy was observed for the F88A^2.53^ mutant, which shows substantially increased cell surface expression compared with wild type, strongly suggests that no receptor reserve is present to affect the observed potency in this assay even at the highest expression levels used in this study.

Examination of the outcomes of such β-arrestin-2 recruitment and interaction studies revealed that mutation of each of five aromatic residues, W104A, F115A^3.29^, F211A^5.42^, W277A^6.48^, and F304A^7.36^, eliminated response to all four ligands (Table 1). Furthermore, mutation of an additional aromatic residue Phe-303^7.35^ to His resulted in a significant (∼10-fold) loss of potency to all four ligands. Expression levels of each of these Ala mutants, and to a lesser extent the F303H^7.35^ mutant, were reduced. However, these levels were similar to those observed with several other mutants that did produce β-arrestin-2 recruitment responses, suggesting that the lack of response represents reduced ligand interaction with FFA4 and not simply reduced mutant expression. To further confirm the importance of these residues, stable Flp-In^TM^ T-REx^TM^ 293 cell lines able to express each of these five mutants were also generated and tested in Ca^2+^ assays (Table 2). These experiments demonstrated that each compound was also inactive at these mutants in the Ca^2+^ assay, with the only exception being a low potency response observed at F304A^7.36^ with TUG-891 only (Table 2). Because the Ca^2+^ assay is predicted to be more sensitive at lower receptor expression levels due to the amplification of its measured response, the lack of responses in this assay strongly supports key roles for these residues in the binding of all four ligands to FFA4. In contrast to the six mutations above that affected the potency of all ligands, five of the initial mutations, L94A^2.59^, M118A^3.32^, T195A, I197A, and V307A^7.39^, were found to have no significant effect on the potency of any of the four ligands tested (Table 1).

As well as the mutations that produced global effects on all ligands tested, ligand-specific effects were also observed within the initial set of mutants. Of particular interest, a group of three mutants, T119A^3.33^, N215A^5.46^, and I280A^6.51^, significantly reduced the potency of the two *ortho*-biphenyl compounds, TUG-891 and TUG-670, while yielding only modest and nonsignificant reductions in potency for GW9508 and not altering the potency of aLA at all when tested in the β-arrestin-2 assay. The T119A^3.33^ and N215A^5.46^ mutations were also assessed in the Ca^2+^ assay (Table 2), which confirmed that these mutations negatively affected the potency of TUG-891 and TUG-670 but not aLA when measured at this end point. However, unlike in the β-arrestin-2 interaction assay where the effect of these mutants did not reach statistical significance, in the Ca^2+^ assay both N215A^5.46^ (*p* < 0.05) and T119A^3.33^ (*p* < 0.001) produced significant reductions in potency for GW9508 compared with wild type FFA4. Together, these results suggest that the T119A^3.33^, N215A^5.46^, and I280A^6.51^ residues are likely critical in forming the binding pocket for the *ortho*-biphenyl fragment and, perhaps to a lesser degree, the pocket for GW9508. There was also a single mutant, I281A^6.52^, that displayed increased ligand potency in the β-arrestin-2 assay however, interestingly, only for TUG-670 and GW9508. This result was again confirmed in the Ca^2+^ assay, also showing significantly increased potency for TUG-670 (*p* < 0.001) and GW9508 (*p* < 0.05) at I281A^6.52^ but not for TUG-891 and aLA (Table 2). That this mutation enhanced the potency of TUG-670, but not TUG-891, suggested the residue might be in close proximity to either the methyl- or fluoro-substituents present in TUG-891 but not TUG-670.

##### Generation of a Refined FFA4-binding Site Model

**FIGURE 3.**
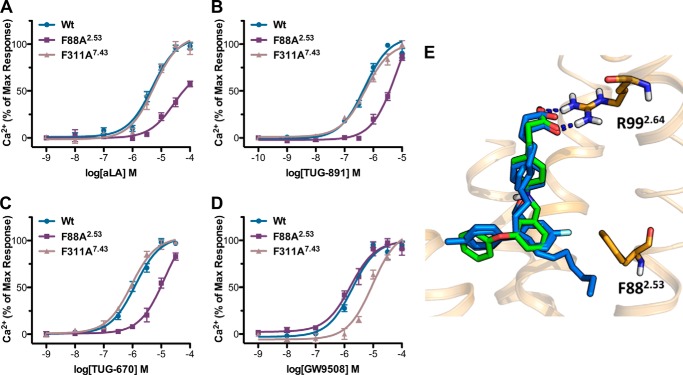
**GW9508 has a distinct mode of binding compared with aLA, TUG-891, and TUG-670.** Ca^2+^ assays show the effect of F88A^2.53^ and F311A^7.43^ mutations on the potency of aLA (*A*), TUG-891 (*B*), TUG-670 (*C*), and GW9508 (*D*). These assays demonstrate that although F88A^2.53^ shows reduced potency for all ligands except GW9508, F311A^7.43^ in contrast displays reduced potency only for GW9508. The binding pose of GW9508 compared with aLA, TUG-891, and TUG-670 is shown in *E*. GW9508 (*green*) is positioned at a longer distance (3.3 Å) from Phe-88 than aLA (2.5 Å), TUG-891 (2.4 Å), and TUG-670 (2.7 Å) (*all blue*) when docked in this model, in agreement with the lack of effect for the F88A^2.53^ mutation on this ligand.

**FIGURE 4.**
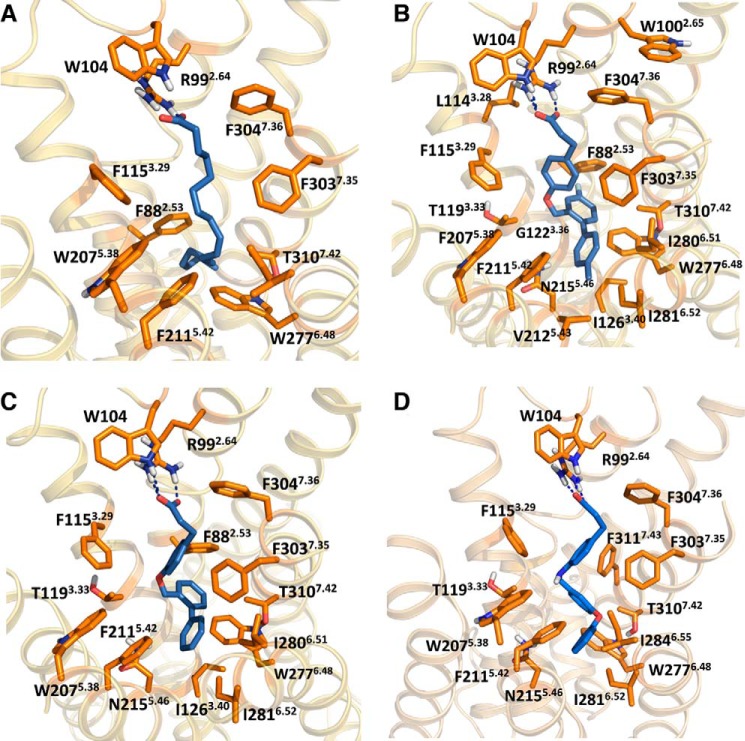
**Orthosteric binding site of FFA4 in complex with aLA, TUG-891, TUG-670, and GW9508.** The binding poses of aLA (*A*), TUG-891 (*B*), TUG-670 (*C*), and GW9508 (*D*) are shown with the side chains of all residues that significantly affect potency when mutated to Ala.

Based on results with the initial set of 21 mutations, a refined binding site model was constructed to identify additional residues potentially involved in ligand binding to FFA4. As all currently described FFA4 ligands are agonists, we aimed at constructing a model that represented the active receptor conformation. Thus, the crystal structure of the nanobody-stabilized β_2_-adrenoreceptor in its active state was chosen as a template for construction of the model. The ligands were docked with flexible binding site residues using the induced fit docking protocol implemented in the Schrödinger Suite. This produced ligand-receptor complexes with the carboxylate interacting with Arg-99^2.64^ without manual intervention, although a constraint was later used at this position to minimize the number of uninteresting poses. aLA and the synthetic ligands docked in a common binding pocket between TM-2, 3, and 5–7 with the carboxylic acid anchored to Arg-99^2.64^ in TM-2. Central to this model were the four aromatic residues F115A^3.29^, F211A^5.42^, W277A^6.48^, and F304A^7.36^ within the TMs that, when mutated, essentially eliminated responsiveness to each ligand tested. These were predicted to line a hydrophobic ligand binding pocket. A further residue, Trp-104, that ablated function of each ligand when it was mutated to alanine is located in the first extracellular loop (ECL1) and was predicted in the refined model to form a hydrogen bond between the indole nitrogen of its side chain and the carboxylate of the ligands. Using this model, an additional seven residues of interest in the receptor were identified, each mutated to Ala, and tested in the β-arrestin-2 interaction assay (and in some cases the Ca^2+^ assay) with aLA, TUG-891, TUG-670, and GW9508 (Table 1, bottom). One of these mutants, W207A^5.38^, resulted in complete loss of function to all ligands, while the T310A^7.42^ mutant also produced a greater than 10-fold reduction in potency to each ligand. The F88A^2.53^ mutant gave a 10-fold reduction in potency for aLA, TUG-891, and TUG-670 but did not significantly affect GW9508, which together with the selective effect of F311A^7.43^ on GW9508 suggests an incomplete overlap with the *ortho*-biphenyl poses. The effects of these two mutants on GW9508 were also confirmed in the Ca^2+^ assay (Table 2), where again F88A^2.53^ significantly reduced potency to aLA, TUG-891, and TUG-670, but not GW9508, while F311A^7.43^ significantly reduced potency only to GW9508 but not the other ligands (Fig. 3, *A–D*). These observations are consistent with the predicted binding pose of GW9508 in the model, suggesting that this ligand is positioned further away from Phe-88 than the other ligands (Fig. 3*C*). The I126A^3.40^ mutant selectively affected the two *ortho*-biphenyl compounds TUG-891 and TUG-670, while V212A^5.43^ reduced potency of TUG-891 only, and both of these effects were also observed in the Ca^2+^ assay (Table 2). I201A and F216A^5.47^ did not result in statistically significant changes in ligand potency. Apart from Trp-207^5.38^ for all four ligands and Trp-100^2.65^ and Leu-114^3.28^ for TUG-891, all mutations that gave a statistically significant change in potency were predicted to be in direct contact with the ligands (Fig. 4, *A–D*). The effect of Trp-207^5.38^ appears to be through stacking with Phe-211^5.42^, positioning this residue correctly toward the general binding pocket.

**FIGURE 5.**
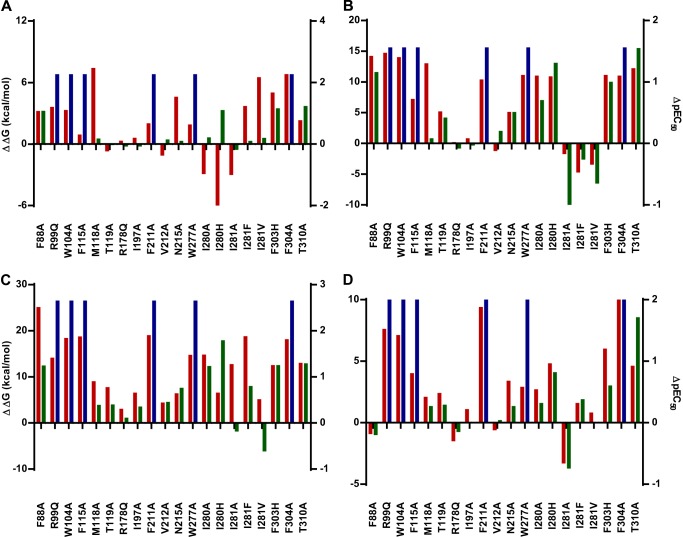
**Comparison of calculated binding energies and experimental potencies obtained for various ligands and mutant forms of FFA4.** Relative effects of mutations on binding energies predicted from docking in mutant receptor models (ΔΔ*G* (kcal/mol), *red bars*, scale on *left side*) and experimentally determined potency (pEC_50_, *green bars*, scale on *right side*, inactive in *blue*) for aLA (*A*) TUG-891 (*B*), TUG-670 (*C*), and GW9508 (*D*).

To validate the refined model, we docked aLA, GW9508, and 10 *ortho*-biphenyl ligands (Table 3) using the extra precision induced fit protocol and calculated relative binding energies by MM-GBSA simulation (see under “Experimental Procedures”). This resulted in good correlation (*r* = −0.84) between pEC_50_ values and calculated relative binding energies (Table 3), indicating that the model explains the binding of the ligands exceptionally well (29). To further test and cross-validate the refined model, we introduced *in silico* mutations to generate individual binding site models of mutants. Binding energies of aLA, TUG-891, TUG-670, and GW9508 in complex with selected mutant models were calculated using MM-GBSA simulation (Table 4). Relative binding energies were generally in very good agreement with the trends observed from the BRET assay (Fig. 5, *A–D*). Considerable loss of binding energy for all four agonists was observed for models incorporating the R99Q^2.64^, W104A, F115A^3.29^, F211A^5.42^, W277A^6.48^, F303H^7.35^, F304A^7.36^, and T310A^7.42^ mutations compared with the wild type FFA4 model, in good agreement with the experimental results. Mutations that resulted in low or nonsignificant reductions in potency in the BRET assay (M118A^3.32^, T119A^3.33^, R178Q, I197A, and V212A^5.43^) were also generally (except M118A^3.32^ for aLA and TUG-670) found to have only small effects on calculated binding energy. The predicted effects of N215A^5.46^ and I280A^6.51^ were satisfactory for the synthetic ligands, but not for aLA, where large negative and positive effects, respectively, were found in the model, compared with nonsignificant experimental effects, suggesting that the pose of the lower part of the fatty acid tail is less accurate. Notably, the specific effect of the F88A^2.53^ mutation on aLA, TUG-891, and TUG-670, but not GW9508, was reflected accurately in the models. Finally, although the predicted binding energies for the I281A^6.52^ mutant suggested TUG-891 should lose potency at this mutant, which was not observed experimentally, the relative effects of modifying the steric bulk at Ile-281^6.52^ through the Ala, Val, and Phe mutations were accurately predicted (see below).

**TABLE 3:**
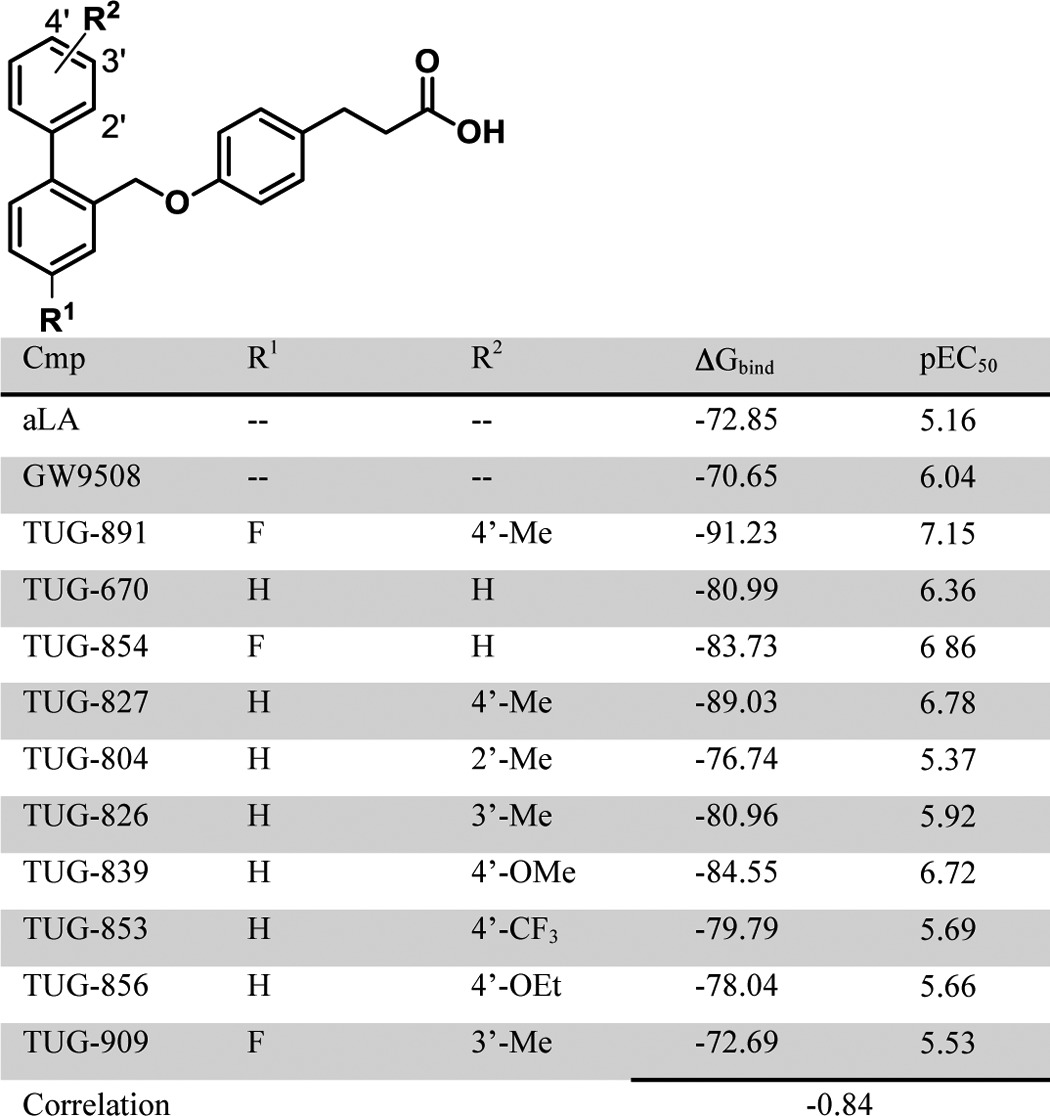
**Calculated binding energy and β-arrestin-2 recruitment potency of various *ortho*-biphenyl compounds**

**TABLE 4:**
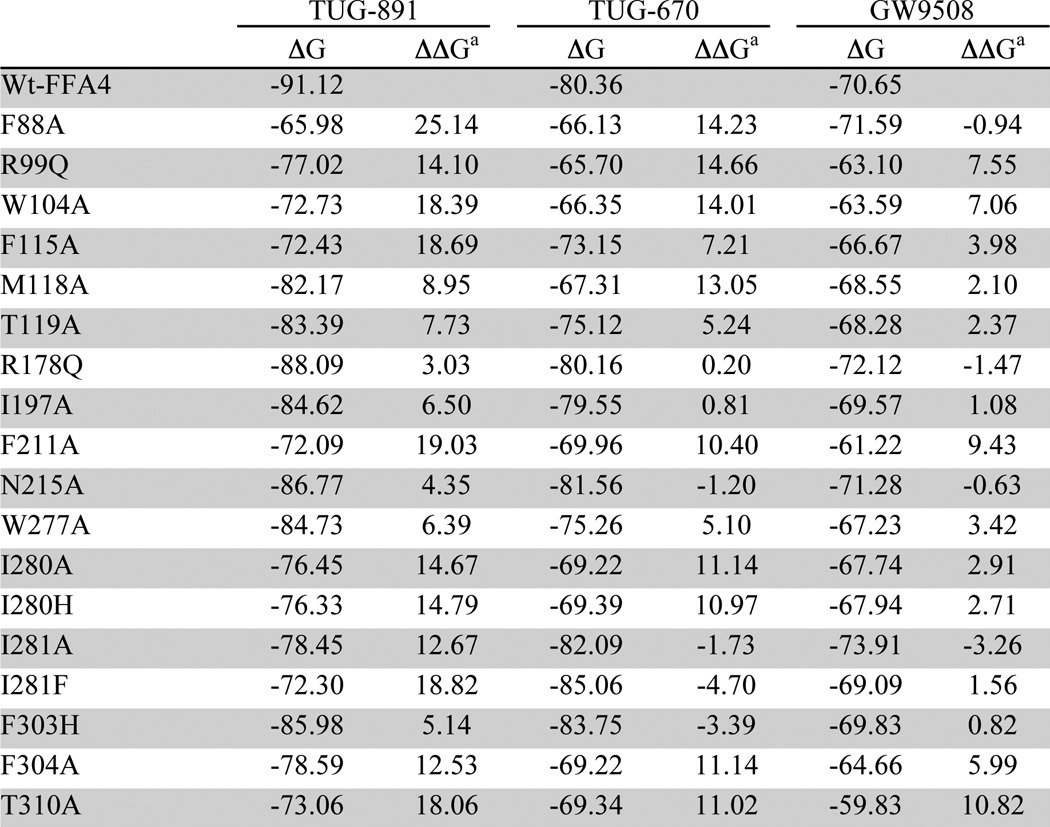
**Calculated binding energies for TUG-891, TUG-670, and GW9508 in models with key residues mutated**

*^a^* ΔΔ*G* is the difference: (Δ*G*_bind_)_mutant_ − (Δ*G*_bind_)_WT_.

The F303H^7.35^ mutation resulted in a significant loss of activity for all ligands. This was reflected in the modeling by a loss of binding energy of the docked ligands in the mutant binding site model as a result of a shift of the hydrophobic phenylpropionate moiety away from the more polar His-303^7.35^. Similar loss of binding energy was observed upon docking of the ligands into models of the F88A^2.53^ and T310A^7.42^ mutants. F88A^2.53^ affects binding energy of TUG-891 and TUG-670 to a larger extent as compared with aLA and GW9508, whereas the T310A^7.42^ mutation affects all the ligands approximately to the same degree. In the cases of T119A^3.33^, N215A^5.46^, and I280A^6.51^, a loss in binding energy was observed only for the *ortho*-biphenyl compounds TUG-670 and TUG-891 but not for aLA or GW9508. Docking in the mutant model V212A^5.43^ caused a moderate loss in binding energy of TUG-891, but it did not affect the other ligands, in agreement with the experimental results.

**FIGURE 6.**
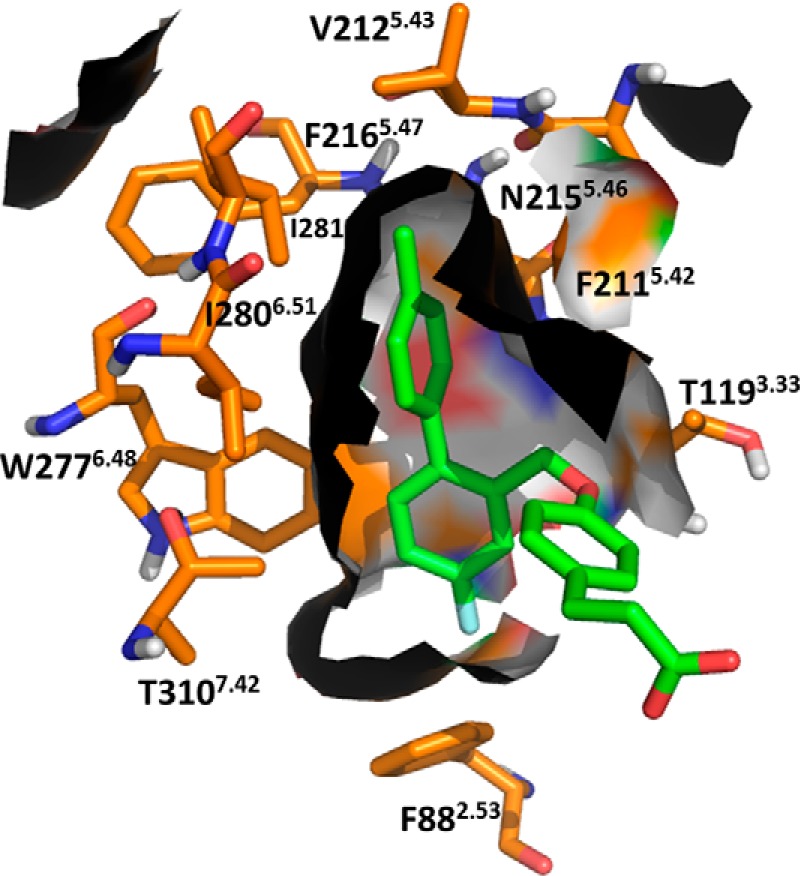
***ortho-*Biphenyl binding pocket of FFA4.** The binding pocket formed by Phe-88^2.53^, Thr-119^3.33^, Gly-122^3.36^, Trp-277^6.48^, Thr-310^7.42^, Asn-215^5.46^, Val-212^5.43^, Phe-211^5.42^, Ile-280^6.51^, and Ile-281^6.52^. Residues are shown in *orange,* and TUG-891 is shown in *green*.

The model suggests combined ionic and hydrogen bond interactions between the ligand carboxylate and the established anchor residue Arg-99^2.64^ and in addition a hydrogen bond interaction with Trp-104 in ECL1. The phenylpropionate moiety of the synthetic ligands interacts with Phe-303^7.35^ and Phe-304^7.36^ on the top side and with Phe-115^3.29^ on the bottom side. The *ortho*-biphenyl part is incorporated into a narrow hydrophobic binding pocket lined by Phe-88^2.53^, Thr-119^3.33^, Gly-122^3.36^, Trp-277^6.48^, Thr-310^7.42^, Asn-215^5.46^, Val-212^5.43^, Phe-211^5.42^, Ile-281^6.52^, and Ile-280^6.51^ (Fig. 6). Supporting this model of TUG-891 binding is the observation that although the Ala mutations of nine of these 10 residues (Ile-281^6.52^ being the one exception) resulted in significant loss of potency of TUG-891, only four significantly lost potency to aLA and only three to GW9508. Indeed, docking of GW9508 to the model suggested a somewhat different mode of interaction within the hydrophobic binding pocket. In particular there appeared to be a critical π-π stacking interaction with Phe-311^7.43^ not present with either TUG-891 or TUG-670 (Fig. 4*D*). This is consistent with the observation that the F311A^7.43^ mutant displayed reduced potency in both β-arrestin-2 and Ca^2+^ assays to GW9508 but not to any of the other ligands tested (Tables 1 and 2).

##### Defining the Role of Isoleucine 281

**FIGURE 7.**
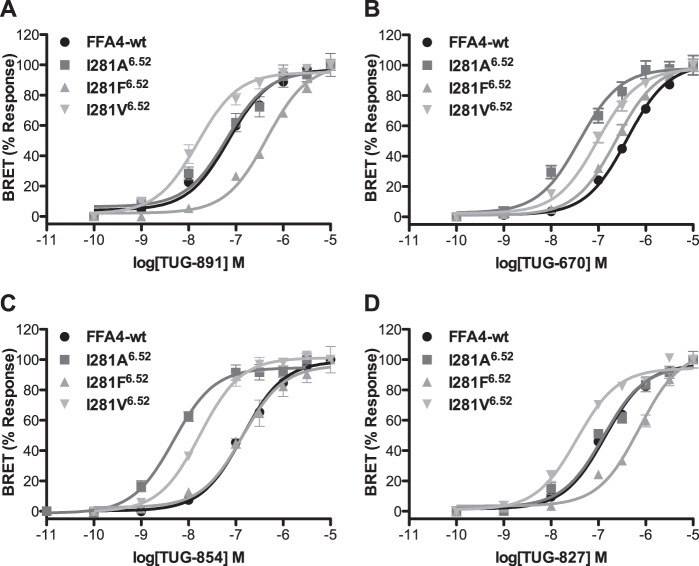
**Effect of Ala, Phe, and Val mutations at position Ile-281^6.52^ of FFA4 on the potency of *ortho*-biphenyl compounds.** Concentration-response curves for TUG-891 (*A*), TUG-670 (*B*), TUG-854 (*C*), and TUG-827 (*D*) at each of wild type (*circles*), I281A^6.52^ (*squares*), I281F^6.52^ (*triangles*), and I281V^6.52^ (*inverted triangle*) FFA4 in the β-arrestin-2 BRET assay.

To examine the predicted mode of binding of TUG-891 in more detail, we next looked at the one mutation predicted to be in the *ortho*-biphenyl binding pocket that did not affect the potency of TUG-891, *i.e.* I281A^6.52^. In the model this residue is located at the top of the *ortho*-biphenyl pocket and is in close proximity to the terminal methyl group of TUG-891. Interestingly, although this mutation did not alter the potency of TUG-891, it did produce an increase in potency of TUG-670 in both β-arrestin-2 and Ca^2+^ assays (Tables 1 and 2). Based on the modeled binding pose, we hypothesized that the difference between TUG-891 and TUG-670 at this mutant might result from the presence of the terminal methyl in TUG-891, which is absent in TUG-670. To test this hypothesis, we examined the potency of various *ortho*-biphenyl analogs at the wild type receptor and the I281A^6.52^ mutant in the β-arrestin-2 assay (Table 5). It was apparent that compounds with a 4′-methyl substituent did not show gain in potency at the I281A^6.52^ mutant, whereas each compound without such a substituent displayed at least a 10-fold increase in potency. Indeed, for compounds with terminal *ortho*- or *meta*-methyl groups, there was a 50-fold increase in potency at the I281A^6.52^ mutant compared with wild type FFA4. To further demonstrate that the terminal methyl of TUG-891 is in close proximity to Ile-281^6.52^, we generated and tested in the β-arrestin-2 assay additional Phe and Val mutations of this residue with *ortho-*biphenyl compounds TUG-891 (Fig. 7*A*), TUG-670 (Fig. 7*B*), TUG-854 (Fig. 7*C*), and TUG-827 (Fig. 7*D* and Table 5). Interestingly, a common trend was again observed, where in this case the I281F^6.52^ mutant reduced the potency of compounds containing the terminal 4′-methyl (TUG-891 and TUG-827), although this alteration had no significant inhibiting or enhancing effect on compounds lacking this methyl group (TUG-670 and TUG-854). In contrast, the I281V^6.52^ mutation resulted in increased potency to all four ligands, although the potency of TUG-670 and TUG-854 at this mutant was not as high as that observed for these compounds with the I281A^6.52^ mutation. Together these results clearly demonstrate that Val at position 281^6.52^ results in optimal binding of compounds containing the terminal methyl, whereas Ala is optimal for compounds lacking this substituent.

**TABLE 5:**
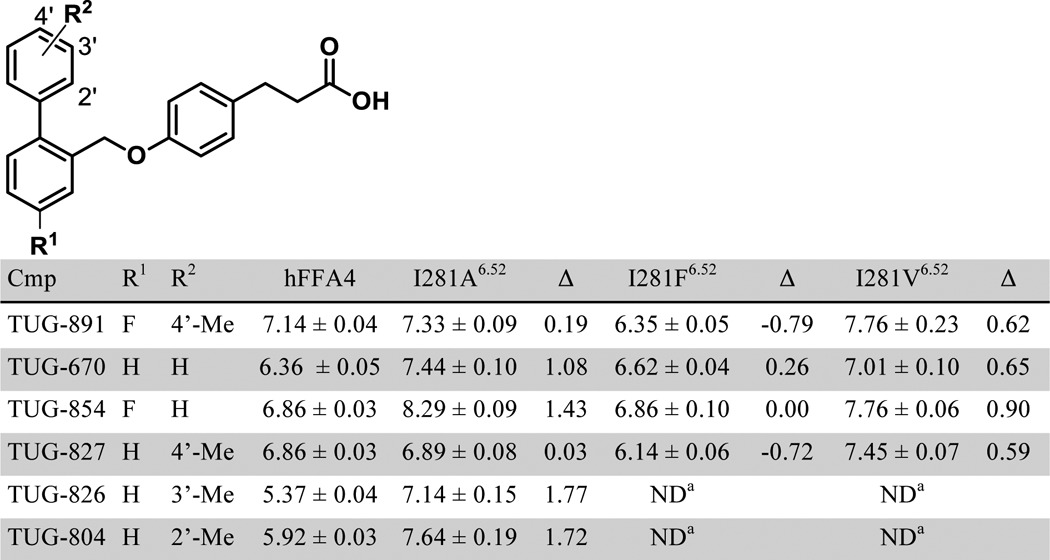
**Effect of Ile-281^6.52^ mutations on β-arrestin-2 recruitment potency for various *ortho*-biphenyl compounds** ND means not determined.

**FIGURE 8.**
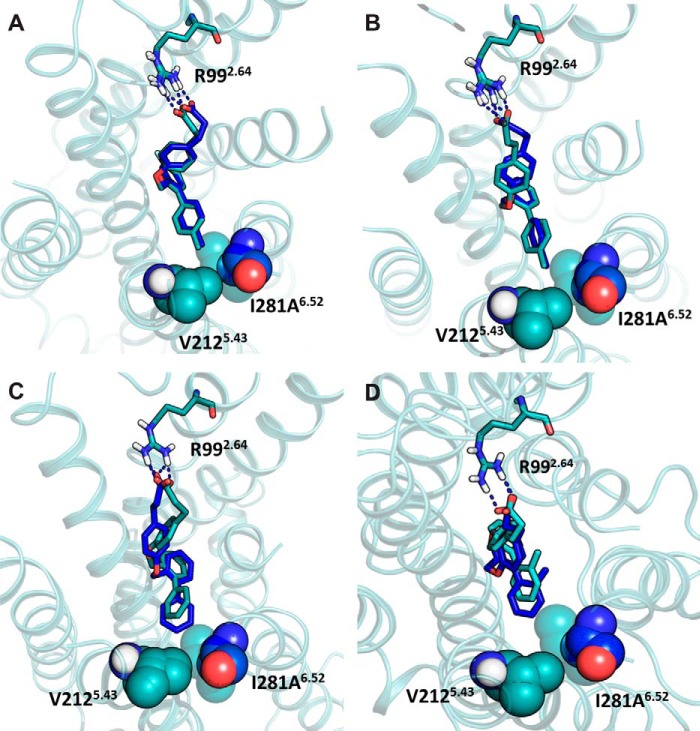
**Effects of the I281A^6.52^ mutation on biphenyl ligands with and without the 4′-methyl substituent.** The poses of TUG-891 (*A*) and TUG-827 (*B*) are minimally affected by the I281A^6.52^ mutation (0.79 and 0.82 Å, respectively), whereas TUG-670 (*C*) and TUG-804 (*D*) shift further into the pocket (1.59 and 1.68 Å, respectively). Ala-281^6.52^ shown as *blue spheres* overlapped by Ile-281^6.52^ shown as transparent *cyan spheres*. The structures docked in the wild type FFA4 model are *cyan*, and structures docked in the I281A^6.52^ mutant model are *blue*.

**FIGURE 9.**
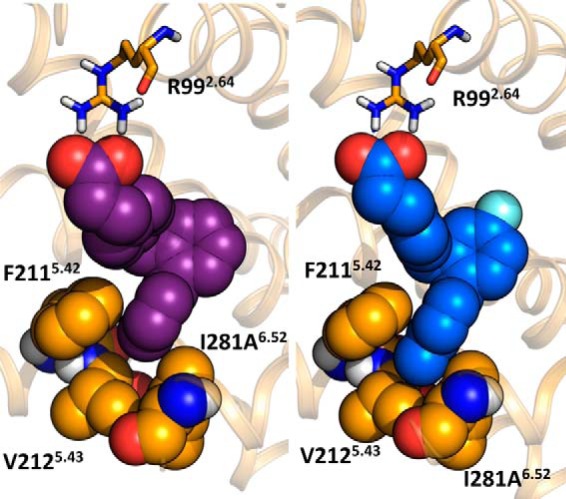
**Terminal 4′-methyl group of TUG-891 is in direct contact with Val-212.** Compounds TUG-670 (*left*) and TUG-891 (*right*) docked in the wild type FFA4. Phe-211^5.42^, Val-212^5.43^, Ile-280^6.51^ (not shown), and Ile-281^6.52^ form a pocket around the terminal ring of the biphenyl system.

Although these experimental results clearly indicate that Ile-281^6.52^ interacts with the terminal methyl of TUG-891, it was still somewhat surprising that the observed effect for the I281A^6.52^ mutant was a gain in potency for compounds lacking this substituent. To examine why this might be the case, we incorporated the Ala mutation into the FFA4-binding site model and explored how this affected the binding pose and predicted binding energy of TUG-891, as well as other *ortho*-biphenyl SAR variants (Table 6). Interestingly, changes in predicted binding energies for these ligands calculated using the model containing Ala-281^6.52^ compared with those calculated using wild type receptor correlated extremely highly (*r* = −0.97) with the experimental potency changes at this mutant (Table 6). Thus, although the model predicted loss of potency for the 4′-methyl analogs TUG-891 and TUG-827 and a more moderate gain of potency than what was observed for the other ligands, the relative changes in calculated binding energy were accurately predicted. Detailed examination of the docking pose in the model incorporating I281A^6.52^ suggests that this mutation allows the top ring of the *ortho*-biphenyl system to shift toward a hydrophobic binding pocket lined by Ile-280^6.51^, Ala-281^6.52^, and Trp-277^6.48^, which leads to better ligand packing and increased potency. This shift in binding pose was restricted for TUG-891 (Fig. 8*A*) and TUG-827 (Fig. 8*B*) by their 4′-methyl substituents, but it was clearly observed for compounds lacking this substituent TUG-670 (Fig. 8*C*) and TUG-854 (Fig. 8*D*), thus accounting for the observation that TUG-891 and TUG-827 did not experimentally gain potency at the I281A^6.52^ mutant. Indeed, the 4′-methyl groups of TUG-891 and TUG-827 were predicted to be in direct contact with Val-212^5.43^ and therefore unable to move further into the binding pocket when it was widened by the I281A^5.43^ mutation (Fig. 9). This prediction is in good agreement with experimental data obtained in both β-arrestin-2 and Ca^2+^ assays demonstrating that V212A^5.43^ loses potency to the 4′-methyl containing TUG-891 but not to TUG-670 (Tables 1 and 2).

**TABLE 6:**
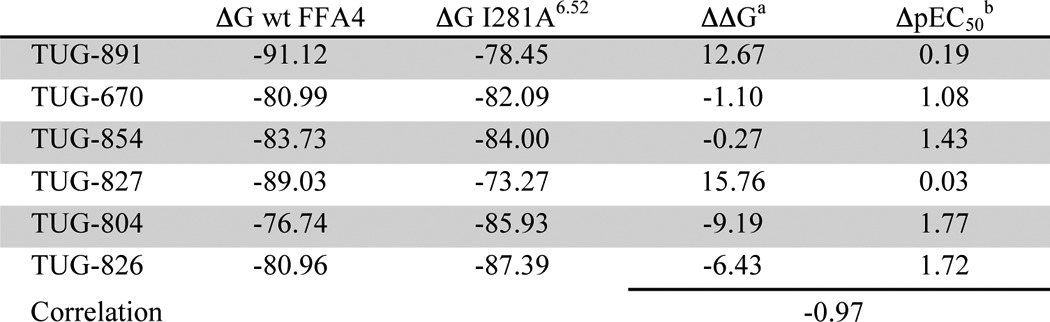
**Calculated binding energy for *ortho*-biphenyl compounds at I281A^6.52^ mutant**

*^a^* ΔΔ*G* is the difference: (Δ*G* I281A^6.52^) − (Δ*G* WT FFA4).

*^b^* Δ pEC_50_ is the difference: (pEC_50_ at I281A^6.52^) − (pEC_50_ at WT FFA4).

## DISCUSSION

FFA4 is a GPCR activated by LCFAs that has garnered significant attention in recent years as a novel therapeutic target for the treatment of metabolic disease (1, 2, 30). Despite this interest, a lack of potent and selective ligands for the receptor has limited its development. Although we recently reported TUG-891 as a potent and selective FFA4 agonist (11), and we used this compound to begin elucidating the potential of this receptor (12), there is clearly still a need for better FFA4 ligands. To facilitate this, this work has conducted the first extensive mutagenesis of FFA4 to define how TUG-891 and other FFA4 agonists bind to this receptor.

Through this, a well validated homology model of FFA4 was constructed that provides a rationale for the molecular basis of ligand interaction with this receptor. Homology modeling has become common practice in structure-based GPCR drug discovery, primarily due to the difficulty obtaining detailed crystal structures of this family of receptors. Although such models have proven useful with other receptors (13, 14), it must also be noted that their accuracy is sometimes questionable. The best example of this being that before the crystal structure of the A_2A_ adenosine receptor bound to its antagonist ligand ZM241385 was published (31), researchers were asked to submit models of this interaction, which were then compared with the obtained crystal structure (32). Of the over 150 models that were examined in this study, very few successfully predicted both the ligand position and ligand-receptor contacts. This highlights the need for good experimental data, as we have provided through both mutational and SAR studies to validate a homology model.

As this is the first detailed examination of the FFA4-binding site, it is also interesting to compare ligand binding with this receptor to how ligands interact with the other LCFA receptor, FFA1, especially because the two receptors are found to recognize the same chemical structures and that obtaining selective compounds indeed can be challenging (11). Previous studies have examined LCFAs and GW9508 binding to FFA1, finding in particular the ligand's carboxylate interacted with Arg-5.39, Asn-6.55, and Arg-7.35, whereas His-3.32, Tyr-3.37, and His-4.56 appeared to form aromatic or hydrophobic interactions specifically with GW9508 (24, 33). Interestingly, none of these sites are conserved in FFA4, and indeed although mutations at positions 6.55 and 7.35 of FFA4 did affect ligand binding, in FFA4 these residues are hydrophobic and therefore do not appear to contribute to ligand binding in the same way as they do in FFA1.

Interestingly, a recent examination of currently available GPCR crystal structures identified trends in the key residues involved in ligand interaction among receptors activated by the same ligand class (15). Although the S1P_1_ receptor is currently the only lipid receptor for which a structure is available (34), it must be pointed out that many of the sites we identify as important for ligand binding to FFA4 are the same as those identified as important in this structure. In particular, residues at 2.64 (Ser in S1P_1_ and Arg in FFA4, both engage in hydrogen bond interactions with the negatively charged group), 5.42 (Phe in both receptors), 6.48 (Trp in both receptors), 6.55 (Leu in S1P_1_ and Ile in FFA4), 6.52 (Phe in S1P_1_ and Ile in FFA4), and 7.39 (Leu in S1P1 and Val in FFA4) were each involved in hydrophobic interactions between the receptor and their respective ligands. Although this perhaps suggests that despite the limited homology between these two receptors their lipid ligands may still interact in a similar manner, clearly this will need to be addressed in more detail in the future.

Finally, it was also interesting to note that there was a clear trend among the four FFA4 agonists tested across all mutations in that the more potent ligands tended to be affected by more mutations. Specifically, although the potency of TUG-891 was altered by mutation at 18 of the 27 residues tested, TUG-670 was only affected by 15, GW9508 by 14, and aLA only by 11. This appears consistent with the prediction that the more potent compounds form more contacts with the receptor, and indeed it is similar to what was previously observed with GW9508 compared with aLA at FFA1 (33).

In recent times the concept of biased signaling of GPCRs, whereby an agonist ligand may selectively activate one signaling pathway over others emanating from the same receptor (for example β-arrestin-mediated *versus* G protein-mediated), has received substantial interest for its potential to yield therapeutics with improved efficacy and reduced adverse effects (35–37). Indeed, given that the anti-inflammatory effects of FFA4 are reported to be β-arrestin-2-mediated (4), and the effects on GLP-1 are likely to be G_q/11_-mediated (3, 12), we have recently developed a G protein-biased form of the FFA4 receptor (38) to help elucidate the potential of biased FFA4 agonists. Considering this, it was important to determine whether the receptor model that was generated in this study based predominantly on results generated in the β-arrestin-2 interaction assay also translated to a G protein-mediated Ca^2+^ end point. This was indeed the case, where each key mutant observed to affect ligand potency in the β-arrestin-2 assay also produced a similar effect in the Ca^2+^ assay. Although this suggests that the model suitably describes a binding pocket that is not biased to favor one of these pathways over the other, this is probably not surprising as we have previously found the ligands studied herein to display little functional bias (11). Indeed, given the general chemical similarity among all currently described FFA4 ligands, comprising a negatively charged carboxylic acid headgroup and an extended hydrophobic tail, there is likely a need for increased chemical diversity among FFA4 agonist ligands before compounds with true bias at this receptor may be identified.

In this work, we have provided the first detailed examination of ligand binding to FFA4. In doing so, we have demonstrated several key residues that when mutated affect ligand function at this receptor, and we have used this information to refine a homology model of the receptor. By using SAR studies in combination with our mutational approach, we have been able to provide strong validation of the mode of ligand binding described in the model. Notably, the model was able to predict the effect of variations or substitution in the biphenyl part of the compound series on the activity on the wild type FFA4 and produced a high correlation between the two parameters. Docking in models of each of 20 mutants also produced results that in the majority of cases predicted the experimentally observed effects. Taken together, this should provide invaluable information to guide future *in silico* drug discovery at the FFA4 receptor.

## Footnotes

4 The abbreviations used are:

GPCR: G protein-coupled receptor
aLA: α-linolenic acid
BRET: bioluminescence resonance energy transfer
eYFP: enhanced yellow fluorescent protein
LCFA: long-chain fatty acid
Rluc: *Renilla* luciferase
SAR: structure-activity relationship
TM: transmembrane
MM-GBSA: molecular mechanics with generalized Born and surface area solvation.
